# Comparison of clinical performance of glass ionomer cement vs. composite resin in restorations of non-carious cervical lesions: A systematic review and meta-analysis

**DOI:** 10.4317/jced.62997

**Published:** 2025-08-01

**Authors:** Heber Isac Arbildo-Vega, Fredy Hugo Cruzado-Oliva, Franz Tito Coronel-Zubiate, Sara Antonieta Luján-Valencia, Joan Manuel Meza-Málaga, Rubén Aguirre-Ipenza, Adriana Echevarria-Goche, Eduardo Luján-Urviola, Tania Belú Castillo-Cornock, Katherine Serquen-Olano

**Affiliations:** 1 Faculty of Dentistry, Dentistry School, Universidad San Martín de Porres, Chiclayo 14012, Peru; 2 Faculty of Human Medicine, Human Medicine School, Universidad San Martín de Porres, Chiclayo 14012, Peru; 3 Faculty of Stomatology, Stomatology School, Universidad Nacional de Trujillo, Trujillo 13001, Peru; 4 Faculty of Health Sciences, Stomatology School, Universidad Nacional Toribio Rodríguez de Mendoza de Amazonas, Chachapoyas 01001, Peru; 5 Postgraduate School, Universidad Católica de Santa María, Arequipa 04013, Peru; 6 Faculty of Dentistry, Dentistry School, Universidad Católica de Santa María, Arequipa 04013, Peru.; 7 Faculty of Biological and Chemical Sciences and Engineering, Universidad Católica de Santa María, Arequipa 04013, Peru; 8 Faculty of Health Sciences, Universidad Continental, Lima 15046, Peru; 9 Faculty of Stomatology, Universidad Peruana Cayetano Heredia, Lima, 15102, Peru; 10 Faculty of Dentistry, Universidad Andina Néstor Cáceres Velásquez, Juliaca 21104, Peru; 11 Faculty of Health Sciences, Stomatology School, Universidad Señor de Sipán, Chiclayo 14000, Peru

## Abstract

**Background:**

To compare the clinical performance of glass ionomer cement (GIC) vs. composite resin (CR) in restorations of non-carious cervical lesions (NCCL).

**Material and Methods:**

A bibliographic search was conducted until October 2023, in the biomedical databases: PubMed/Medline, Cochrane Library, SciELO, Scopus, Web of Science and Google Scholar. Randomized clinical trials reporting the effect of GIC compared to CR in the restoration of NCCLs were included, without restrictions on publication date or language. The RoB 2.0 tool was used to assess the risk of bias of the included studies and the GRADEPro GDT tool was used to assess the quality of evidence and the strength of recommendations.

**Results:**

The search yielded a total of 296 articles. After excluding those that did not meet the selection criteria, 18 articles remained for the quantitative synthesis. The analysis found no statistically significant differences between CR and GIC in the restoration of NCCLs.

**Conclusions:**

The literature reviewed suggests that there are no differences in clinical performance over time when restoring NCCLs with CRs or GICs.

** Key words:**Non-carious cervical lesion, composite resin, glass ionomer cement, review, meta-analysis.

## Introduction

Non-carious cervical lesion (NCCL) restorations are a key component in restorative dentistry, and choosing the appropriate restorative material is essential to achieving long-term clinical success [1]. In this context, the comparison between glass ionomer cement (GIC) and composite resin (CR) has hained increasing relevance in dental research and practice, since both materials exhibit unique properties that influence their effectiveness and longevity for the treatment of NCCL [2].

The worldwide prevalence of NCCL, especially in older populations in South America, is significant [3]. These lesions, which are characterized by the loss of dental tissue in the cervical region, are associated with diverse factors - including abrasion, erosion, abfraction, and attrition – making their management clinically complex [4-6]. Abfraction, which involves the microstructural loss of hard dental tissue, is a common manifestation of NCCLs and its multifactorial aetiology has been the subject of study, with factors such as occlusal forces and eccentric occlusal loading playing a relevant role in its development [7,8].

In particular, the choice of restorative material, whether traditional glass ionomer cement or composite resins, has a significant impact on the success of NCCL restorations, considering adhesive properties, fluoride release, aesthetics and mechanical properties [6]. The literature has explored these variables through diverse approaches, including adhesive strategies, diode laser treatments and evaluations of restoration retention, generating important yet scattered findings [9-13]. Likewise, due to the complications of NCCL, restorative treatment has been combined with some surgical techniques [14], where the assessment of dental restorations becomes even more important.

The need for a systematic review and meta-analysis arises from the lack of a com-prehensive synthesis of the evidence, which would allow dental professionals to make clinical decisions based on concrete data. Therefore, this study aims to address this gap in the literature by critically evaluating the clinical performance of the two most used restorative materials (GIC and CR) in the context of NCCLs, to provide consolidated evidence and guide clinical practice.

## Material and Methods

1. Protocol

The present review was conducted based on the Preferred Reporting Items for Systematic Reviews and Meta-Analyses Protocols (PRISMA-P) [15] and registered in the Prospective Registry of Systematic Reviews (PROSPERO) [16]. The registry is publicly available under CRD number 42023472658.

The focused question was formulated using the PICO format (population, intervention, outcomes and results) as detailed below:

- Population: People with non-carious cervical lesions and without systemic diseases

- Intervention: Restoration of non-carious cervical lesions with GIC (conventional and/or resin-modified)

- Comparison: Restoration of non-carious cervical lesions with CR

- Outcomes: Secondary caries, marginal discoloration, marginal adaptation, marginal integrity, colour or translucency, surface texture or luster, surface staining, retention, wear, anatomical form, sensitivity and state of periodontal tissues

2. Focused question (PICO)

Is there a difference in clinical performance between glass ionomer and composite resin in the restoration of non-carious cervical lesions?

3. Search and selection of studies

A comprehensive literature search was conducted in five electronic databases (PubMed/Medline, Cochrane Library, Scopus, Web of Science and Scielo). Gray literature was also consulted through Google Scholar, OpenGrey and Proquest. Additionally, the reference lists of included studies were reviewed; covered all publications up to April 2025; combining keywords and subject titles according to the thesaurus of each database: “Root caries”, “Cervical caries”, “Non carious cervical injury”, “Non-carious cervical injury”, “Cervical injury”, “Cervical injuries” , “Glass Ionomer Cements”, “Glass-Ionomer Cement”, “GIC”, “GICs”, “Composite resin”, “Resin composite”, “Composite resin”, “Resin compomer”, “Clinical trial” and “Randomized clinical trials”. The search strategies for each of the databases are found in  Table 1.

Additionally, further relevant literature was included after a hand search of the reference lists of the final included articles.

The initial screening of titles and abstracts was conducted independently by two authors (HA and FCO), and the final inclusion decision was made according to the following criteria: Randomized clinical trials (RCTs), without time and language limits and reporting the effect of GIC and CR in restorations of NCCLs. Articles that were prospective studies, unpublished studies and reported in more than one publication with different follow-up periods were excluded.

4. Data extraction 

Data were extracted using a standardized Table that included the following variables: author(s), year of publication, study design, country where the study was conducted, number of patients, proportion of male and female patients, age mean and age range, follow-up time, evaluation criteria, study groups, number of patients and teeth restored per study group, secondary caries, marginal discoloration, marginal adaptation, marginal integrity, colour or translucency, surface texture or luster, surface staining, retention, wear, anatomical form, sensitivity and periodontal tissues. From each eligible study, two investigators (FCZ and SL) independently extracted information and all disagreements were resolved by discussion with a third reviewer (JM).

5. Risk of bias (RoB) assessment

The RoB of the included studies was independently assessed by two calibrated authors (RA and AE) (k = 0.98) using the Cochrane RoB 2.0 tool [17] and all disagreements were resolved by discussion with a third reviewer (EL). According to this tool, clinical trials are evaluated in 5 domains: randomization process, deviations from planned interventions, missing outcome data, outcome measurement, and selection of the results report; to later be classified as: high risk of bias, bias with some concerns or low risk of bias.

6. Analysis of results

All statistical analyses were performed using Review Manager (RevMan) version 5.3 (Cochrane Collaboration, UK); using the mean and standard deviation and frequency as a measure, in a random effects model with a 95% confidence interval. Additionally, the certainly of evidence and strength of recommendations were assessed using the GRADE approach via GRADEPro GDT (McMaster University and Evidence Prime Inc., Canada).

## Results

**Figure 1:**
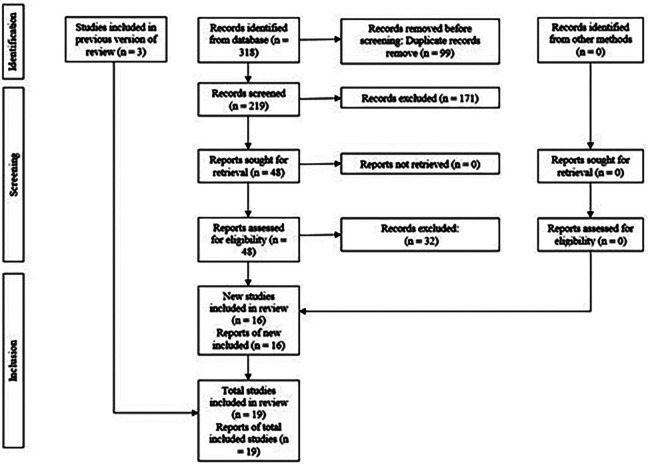
PRISMA diagram showing the process of inclusion and exclusion of studies.

1. Selection of studies:

The electronic and manual search strategy yielded 318 articles. After removing 99 duplicates (Fig. 1), 171 were excluded during title screening, leaving 48 potentially eligible for abstracts screening, 32 articles were excluded, and 3 additional studies were included through manual search of other reviews, resulting in 19 RCTs that met the eligibility for qualitative and quantitative synthesis (meta-analysis).

During the screening process, several full-text articles were excluded for specific reasons related to the eligibility criteria (Glass ionomer was used as an adhesive system, non-glass ionomer group, non-composite resin group, polyacid modified resin group, patients with systemic disease, study with data reported in another publication with different follow-up period, fluid resin group, non-randomized clinical trials). A detailed list of these excluded studies, along with the reasons for their exclusion, is provided in (Supplement 1) (http://www.medicinaoral.com/medoralfree01/aop/jced_62997_s01.pdf).

2. Characteristics of included studies

In total, 19 RCTs [18-36] were included, of which only one [22] was parallel. All studies reported that the total number of patients ranged from 10 to 88 with the number of teeth treated ranging from 16 to 170. Ten studies [18,19,22,24,25,28,29,31,33,36] reported that the mean age of patients ranged from 47 to 62.2 years, and all studies reported with age ranges between 18 and 92 years with a follow-up time of between 3 months and 10 years (Supplement 2) (http://www.medicinaoral.com/medoralfree01/aop/jced_62997_s02.pdf).

The countries where the studies were carried out were: Egypt (20), India [21,25], Brazil [19,27,28,30], Germany [22,34,35], Turkey [18,24,32], Vietnam [23], Romania [26], Nigeria [29], Australia [31], Mexico [33] and the United States [36]. Three studies [22,24,25] mentioned that the evaluation criteria used for the analysis of the teeth was the FDI criteria. Two studies [22,24] reported the use of high viscosity glass ionomer cement (HVGIC), one study [36] used glass ionomer cement (GIC), while the remaining studies used resin-modified glass ionomer cement (RMGIC) (Supplement 2) (http://www.medicinaoral.com/medoralfree01/aop/jced_62997_s02.pdf).

In sixteen [18-22,24-30,32,33,35,36], seventeen [18–22,24-30,32-36], twelve [18,20,22-29,33,36], seven [19,21,24,30,32,34,35], eleven [19,22-24,26-28,32-35], ten [19,22-24,28,32-36], four [19,22,24,36), eighteen [18-34,36), six [19,22,24,28,29,36], thirteen [19,21-24,26,27,30,32-36], seven [18,20,22,24,25,28,29] and three [19,23,24] studies reported on secondary caries, marginal discoloration, marginal adaptation, marginal integrity, colour or translucency, surface texture or luster, staining surface, retention, wear, anatomical shape, sensitivity and state of periodontal tissues, respectively ( Table 2).

(Supplement 2) (http://www.medicinaoral.com/medoralfree01/aop/jced_62997_s02.pdf).

3. Risk of bias analysis of studies

**Figure 2:**
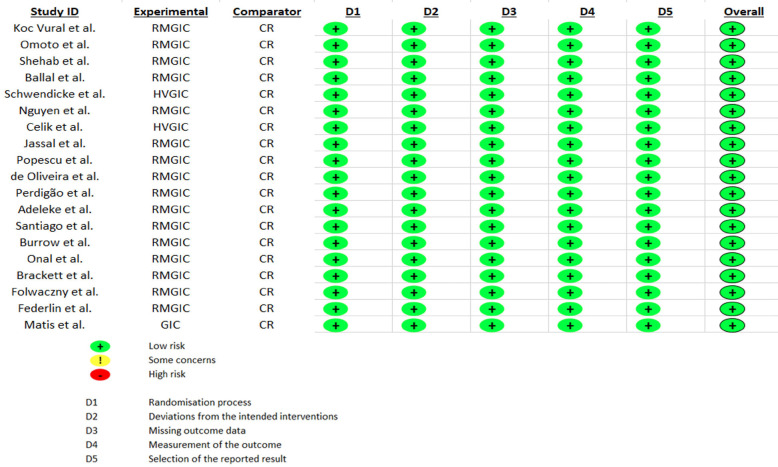
Risk of bias analysis of included studies.

All studies had a low risk of bias (Fig. 2).

Fig. 2. Risk of bias analysis of included studies

4. Synthesis of results (Meta-analysis)

The clinical performance of GIC in comparison to CR in terms of absence of secondary caries, absence of marginal discoloration, adequate marginal adaptation, adequate marginal integrity, adequate color or translucency, proper surface texture or luster, proper surface staining, retention, absence of wear, proper anatomic form, absence of sensitivity and adequate periodontal tissue was determined in sixteen [18-22,24–30,32,33,35,36], seventeen [18-22,24-30,32-36], twelve [18,20,22-29,33,36], seven [19,21,24,30,32,34,35], eleven [19,22-24,26-28,32-35], ten [19,22-24,28,32-36], four [19,22,24,36], eighteen [18-34,36], six [19,22,24,28,29,36], thirteen [19,21-24,26,27,30,32-36], seven (18,20,22,24,25,28,29] and three [19,23,24] studies; revealing no statistically differences for any of these clinical parameters. Regarding heterogeneity, most clinical outcomes showed low to moderate heterogeneity (I² < 50%). However, higher heterogeneity was observed in marginal integrity (I² = 71%), surface texture or luster (I² = 69%), and retention (I² = 73%). These differences could be attributed to clinical diversity among studies, including variation in lesion characteristics (depth, tooth location), material brands, operator techniques, patient oral hygiene, and follow-up durations. Full heterogeneity values for each outcome are detailed in (Supplement 3) (http://www.medicinaoral.com/medoralfree01/aop/jced_62997_s03.pdf). (Figures C1 – C12).

Publication bias was assessed using funnel plots and Egger´s regression test for all 12 clinical parameters (Appendix C, Figures C1 – C12). A statistically significant asymmetry suggesting potential publication bias was observed for the “retention” parameter (Egger´s test *p* = 0.006). For all other outcomes, Egger´s test indicated no significant publication bias (*p* > 0.05).

5. Subgroup synthesis

The clinical performance of GIC compared to CR in terms of absence of secondary caries, absence of marginal discoloration, adequate marginal adaptation, adequate marginal integrity, adequate colour or translucency, proper surface texture or luster, proper surface staining, retention, absence of wear, proper anatomic form, absence of sensitivity and adequate periodontal tissue, in relation to the different follow-up periods; it was determined in fifteen [18-22,24-30,32,33,35], sixteen [18-22,24-30,32-35], ten [18,20,22,24-29,33], seven [19,21,24,30,32,34,35], ten [19,22,24,26-28,32-35], eight [19,22,24,28,32–35), three [19,22,24], seventeen [18-34), five [19,22,24,28,29], eleven [19,21,22,24,26,27,30,32-35), seven [18,20,22,24,25,28,29] and two [19,24] studies. It was shown that there was no statistically significant difference for all these clinical parameters. In subgroup analyzes based on follow-up duration, high heterogeneity (I² > 75%) was noted in several outcomes such as marginal discoloration (up to I² = 92%), marginal integrity (up to I² = 94%), and retention (up to I² = 90%). These results may reflect greater variability in short- to medium-term clinical performance, differences in restorative protocols, and fewer studies included per subgroup. These values are reported in detail in (Supplement 3) (http://www.medicinaoral.com/medoralfree01/aop/jced_62997_s03. (Figures C13 – C24).

Publication bias was also evaluated for subgroups analyses (Supplement 3) (http://www.medicinaoral.com/medoralfree01/aop/jced_62997_s03., Figures C13 – C24). Statistically significant Egger´s test results (*p* < 0.05) were observed in the following subgroup comparisons: marginal discoloration at 3 years (*p* = 0.003), marginal integrity at 6 months (*p* = 0.01), color/translucency at 1.5 years (*p* = 0.002), retention at 1, 2, and 3 years (*p* = 0.02, 0.00, 0.00), and anatomic form at 6 months (*p* = 0.04), suggesting possible publication bias. These findings should be interpreted with caution, particularly for subgroup analyses including fewer than 10 studies.

6. GRADE analysis

When evaluating the included studies, it was observed that there is high certainty in the absence of secondary caries, adequate marginal adaptation, adequate colour or translucency, proper surface staining, absence of wear, absence of sensitivity and adequate periodontal tissues; there is moderate certainty in the absence of marginal discoloration, adequate marginal integrity and proper anatomic form. In contrast, there is low certainty in proper surface texture or luster and retention ( Table 2).

## Discussion

In the present study, we found that the clinical performance of GIC and CR was similar across all evaluated parameters (secondary caries, marginal discoloration, marginal adaptation, marginal integrity, surface color, wear, hypersensitivity, color or translucency, surface texture, retention, periodontal tissue, and anatomical shape) for restoring NCCL. Meta-analyses were conducted to each parameter at 6, 12, 18, 24 and 36 months of follow-up, allowing for a comprehensive evaluation of performance over time. To determine the overall strength of evidence, a GRADE analysis was performed. It showed high certainty for secondary caries, marginal adaptation, color/translucency, surface staining, wear, sensitivity, and periodontal tissue; moderate certainty for marginal discoloration, marginal integrity, and anatomic form; and low certainty for surface texture and retention.

Among the 18 studies included, only one evaluated conventional GIC [36], while two assessed HVGIC [22,24). As a result, the findings mainly reflect the performance of resin-modified GIC (RMGIC), which was the predominant type of material evaluated. RMGICs were developed to overcome certain limitations of conventional GIC, including esthetic deficiencies, early moisture sensitivity, and reduced mechanical strength [18-21,23,25-35].

According to the literature, one of the primary challenges in NCCL restorations is achieving proper retention. The lesions, in most cases, are small, shallow and contain dentin tissue with sclerosis at the level of the tubules, which is a tissue with increased mineralization that impairs adhesive bonding [37]. In our review, which evaluated the retention factor in the short (6 months), medium (18 months) and long term (36 months), there were no differences between the materials. This may be due to the similar bonding mechanisms of both materials to the tooth structure. In addition to their micromechanical retention, both have the potential for chemical bonding to the tooth, interacting superficially with the dentin and without completely dissolving hydroxyapatite crystals around the collagen matrix [25]. In contrast to findings of Bezerra *et al*. [2] and Boing *et al*. [37], where this parameter presented a difference.

The adaptation of the restorative material is influenced by the high modulus of elasticity that these materials present, which could enhance resistance to deformation, which possibly contributes considerably to their marginal adaptation [26]. On the other hand, adhesion at the level of the NCCL is compromised due to the lack of enamel, which makes good marginal integrity difficult. It may be due to thermal and mechanical stresses in the oral environment, viscoelastic properties of the restorative material, water absorption, hydrolysis, and unique stress patterns at the cervical margin of the tooth. Shrinkage stresses create forces that compete with the adhesive bond and potentially disrupting the adhesive interface to the cavity walls [27]. The formation of marginal spaces and excesses on the margins of the restoration influence marginal discoloration [36]. However, the ability of adhesives to seal microporosities and modern finishing and polishing techniques minimize this parameter [25]. Likewise, these mechanisms contribute to the absence of secondary caries and good periodontal health, when combined with proper oral hygiene practices [38].

In the oral cavity, these materials endure stress due to cyclic bending deformation in the cervical region during occlusal loading of the tooth, which can cause fatigue cracks. Due to the propagation of these cracks, material loss through abfraction may eventually occur, which is then interpreted as wear of the anatomical shape of the restoration [34]. In addition, this material is constantly exposed to wear due to abrasion and erosion throughout its time in the oral cavity [36]. With the modification of the components of the GIC to RMIGC and the new CR, their wear resistance has improved. In the same way, modifying the size of the filler particles of the organic component results in a better texture of the surface of the restoration [35].

Postoperative tenderness after NCCL treatment is a common problem encountered in a clinical setting. Increased postoperative sensitivity from the patient’s perspective would be an undesirable outcome [39]. With new self-etching adhesive systems and selective etching technique, it causes the smear layer to dissolve, incorporating it into the mixture of collagen fibres and resin monomers, resulting in the smear layer becoming an integral part of the hybrid layer, reducing or eliminating postoperative sensitivity [40]. Furthermore, the sensitivity phenomenon gradually decreases over time as a result of tertiary dentin formation after restoration [23].

Finally, the stability of the surface colour maintained over time can be explained, among other factors, by the adequate polymerization of the restorative materials, since incomplete polymerization and the presence of residual monomers can cause an alteration of the colour through an increase in the sorption of oral fluids, dyes, good finish and polishing performed [27].

While publication bias was not detected in most comparisons, statistically asymmetry was identified in specific parameters, particularly retention in the min analysis and multiple subgroup outcomes. These findings may reflect selective reporting, small-study effects, or other sources of bias. It is important to interpret these subgroup results with caution, especially when the number of studies was limited, which can affect the reliability of Egger´s test and funnel plot interpretations.

Regarding heterogeneity, although several outcomes such as secondary caries, marginal adaptation, colour, wear, sensitivity and periodontal tissue showed low heterogeneity (I² = 0%), other parameters such as marginal integrity (I² = 71%), surface texture or luster (I² = 69%) and retention (I² = 73%) showed substantial heterogeneity. These differences may reflect clinical and methodological variability across studies, such as different follow-up periods, operator skill, lesion location and severity, restorative material brand, and evaluation criteria. Subgroup analyzes further revealed very high heterogeneity in some outcomes (I² > 90%), particularly in early timepoints and for esthetic parameters. These limitations should be considered when interpreting the findings of our meta-analyses.

From a clinical standpoint, the comparable performance of GIC and CR in restoring NCCLs allows for a more individualized approach to treatment planning. GIC may be favored in patients with high caries risk due to its fluoride-releasing capability and chemical adhesion, while CR may be preferred in aesthetically demanding areas due to superior polishability and shade matching. The absence of significant differences supports the notion that clinicians can base their material selection on context-specific factors rather than perceived superiority. However, awareness of the limitations in evidence quality for certain parameters—such as retention and surface texture—remains critical when counseling patients and selecting materials.

The similarity of the clinical materials analyzed in this systematic review and meta-analysis suggests that both GIC and CR exhibit comparable and clinically accepTable outcomes. However, they should be interpreted with caution, as this summarized evidence included studies con-ducted under different conditions. More recent clinical studies are likely to evaluate recently launched products. However, limited evidence is currently available regarding these newer formulations and more well-designed clinical trials are needed to evaluate them. This systematic review considered the full range of GICs and CRs reported in the literature, regardless of which brand was used. Furthermore, the mechanical, biological and optical properties of materials can vary substantially among the different formulations of GIC and CR available on the market.

These results have important clinical implications. The finding that both GIC and CR show comparable performance in NCCL restorations allows clinicians greater flexibility in material selection. For example, in patients with a high caries risk or limited moisture control, GIC may be favored due to its fluoride release and chemical bonding potential. Conversely, CR may be preferred in aesthetically demanding cases, particularly in anterior regions. Therefore, the material choice can be guided by patient-specific needs, clinical environment, and operator preference without compromising clinical longevity.

Limitations

This review has several limitations. First, the included studies used a wide variety of glass ionomer and composite resin materials, with potential differences in composition, handling properties, and clinical performance that were not stratified in the analysis. Second, the substantial heterogeneity in some outcomes may affect the precision and generalizability of the findings. Although the random-effects model was used, residual confounding due to differences in lesion characteristics, evaluation methods, operator variability, and patient-related factors may persist. Third, publication bias was detected in some analyzes (e.g., retention), suggesting a potential overrepresentation of favorable outcomes. Finally, although only randomized clinical trials were included, not all studies had identical follow-up periods or criteria, which could influence comparability.

## Conclusions

According to the findings of the present review, there are no significant differences in the clinical performance or durability when restoring NCCLs with composite resins or glass ionomer cements. These findings support the clinical use of either material, allowing for individualized treatment decisions based on esthetic demands, moisture control, and patient caries risk. However, the results should be interpreted with caution due to the high heterogeneity observed in some parameters and the moderate to low quality of evidence for specific outcomes.

## Figures and Tables

**Table 1:** Database search strategy.

Database	Search strategy	Number of study
Pubmed	(("Root Caries") OR ("Cervical Caries") OR ("Non carious cervical lesion") OR ("Non-carious cervical lesion") OR ("Cervical lesion") OR ("Cervical lesions")) AND (("Glass Ionomer Cements") OR ("Glass-Ionomer Cement") OR ("GIC") OR ("GICs")) AND (("Composite resin") OR ("Resin composite") OR ("Composite resin") OR ("Resin compomer")) AND (("Clinical trial") OR ("Randomized clinical trial"))	34
Cochrane Library	#1 MeSH descriptor: [Root Caries] explode all trees #2 ("Root Caries") OR ("Cervical Cary") OR ("Cervical Caries") OR ("Non carious cervical lesion") OR ("Non-carious cervical lesion") OR ("Cervical lesion") OR ("Cervical lesions") (Word variations have been searched) #3 #1 OR #2 #4 MeSH descriptor: [Glass Ionomer Cements] explode all trees #5 ("Glass ionomer cements") OR ("Glass-Ionomer Cement") OR ("GIC") OR ("GICs") (Word variations have been searched) #6 #4 OR #5 #7 MeSH descriptor: [Composite Resins] explode all trees #8 ("Composite resin") OR ("Resin composite") OR ("Composite resin") OR ("Composite resins") OR ("Resin composites") OR ("Resin compomer") (Word variations have been searched) #9 #7 OR #8 #10 #3 AND #6 AND #9	59
Scielo	(("root caries") OR ("Cervical Caries") OR ("Non carious cervical lesion") OR ("Non-carious cervical lesion") OR ("Cervical lesion") OR ("Cervical lesions")) AND (("Glass Ionomer Cements") OR ("Glass-Ionomer Cement") OR ("GIC") OR ("GICs")) AND (("Composite resin") OR ("Resin composite") OR ("Composite resin") OR ("Resin compomer"))	0
Scopus	(TITLE-ABS-KEY ("Root Caries") OR TITLE-ABS-KEY ("Cervical Cary") OR TITLE-ABS-KEY ("Cervical Caries") OR TITLE-ABS-KEY ("Non carious cervical lesion") OR TITLE-ABS-KEY ("Non-carious cervical lesion") OR TITLE-ABS-KEY ("Cervical lesion") OR TITLE-ABS-KEY ("Cervical lesions")) AND (TITLE-ABS-KEY ("Glass ionomer cements") OR TITLE-ABS-KEY ("Glass-Ionomer Cement") OR TITLE-ABS-KEY (GIC) OR TITLE-ABS-KEY (GICs)) AND (TITLE-ABS-KEY ("Composite resin") OR TITLE-ABS-KEY ("Resin composite") OR TITLE-ABS-KEY ("Composite resin") OR TITLE-ABS-KEY ("Composite resins") OR TITLE-ABS-KEY ("Resin composites") OR TITLE-ABS-KEY ("Resin compomer")) AND (TITLE-ABS-KEY ("Clinical trial") OR TITLE-ABS-KEY ("Randomized clinical trial")) AND (LIMIT-TO (DOCTYPE, "ar"))	47
Web of Science	(TS=("Root Caries") OR TS=("Cervical Cary") OR TS=("Cervical Caries") OR TS=("Non carious cervical lesion") OR TS=("Non-carious cervical lesion") OR TS=("Cervical lesion") OR TS=("Cervical lesions")) AND (TS=("glass ionomer cements") OR TS=("Glass-Ionomer Cement") OR TS=(GIC) OR TS=(GICs)) AND (TS=("Composite resin") OR TS=("Resin composite") OR TS=("Composite resin") OR TS=("Composite resins") OR TS=("Resin composites") OR TS=("Resin compomer"))	54
Google Scholar	("Cervical Caries" OR "Non carious cervical lesion" OR "Non-carious cervical lesion" OR "Cervical lesion") + ("Glass Ionomer Cement" OR "Glass-Ionomer Cement" OR GIC OR GICs) + ("Composite resin" OR "Resin composite" OR "Composite resin" OR "Resin compomer") -"in vitro" -"systematic review"	121
Open Gray	(("Root Caries") OR ("Cervical Caries") OR ("Non carious cervical lesion") OR ("Non-carious cervical lesion") OR ("Cervical lesion") OR ("Cervical lesions")) AND (("Glass Ionomer Cements") OR ("Glass-Ionomer Cement") OR ("GIC") OR ("GICs")) AND (("Composite resin") OR ("Resin composite") OR ("Composite resin") OR ("Resin compomer")) AND (("Clinical trial") OR ("Randomized clinical trial"))	0
Proquest	("Root Caries" OR "Cervical Caries" OR "Non carious cervical lesion" OR "Non-carious cervical lesion" OR "Cervical lesion" OR "Cervical lesions") AND ("Glass Ionomer Cements" OR "Glass-Ionomer Cement" OR "GIC" OR "GICs") AND ("Composite resin" OR "Resin composite" OR "Composite resin" OR "Resin compomer") AND ("Clinical trial" OR "Randomized clinical trial") NOT ("systematic review" OR "in vitro" OR "review")	6

**Table 2:** GRADE analysis of included studies.

Certainty assessment							of patients		Effect		Certainty
of studies	Study design	Risk of bias	Inconsistency	Indirectness	Imprecision	Other considerations	GIC	CR	Relative (95% CI)	Absolute (95% CI)	
Absence of secondary caries (follow-up: range 1 years to 10 years)											
16	randomized trials	not serious	not serious	not serious	not serious	none	640/645 (99.2%)	569/574 (99.1%)	RR 1.00 (0.99 to 1.01)	0 fewer per 1,000 (from 10 fewer to 10 more)	High
Absence of marginal discoloration (follow-up: range 1 years to 10 years)											
17	randomized trials	not serious	serious	not serious	not serious	none	642/709 (90.6%)	602/650 (92.6%)	RR 1.00 (0.97 to 1.02)	0 fewer per 1,000 (from 28 fewer to 19 more)	Moderate
Adequate marginal adaptation (follow-up: range 3 months to 10 years)											
12	randomized trials	not serious	not serious	not serious	not serious	none	525/558 (94.1%)	495/527 (93.9%)	RR 1.00 (0.99 to 1.02)	0 fewer per 1,000 (from 9 fewer to 19 more)	High
Adequate marginal integrity (follow-up: range 1 years to 4 years)											
7	randomized trials	not serious	serious	not serious	not serious	none	218/245 (89.0%)	205/219 (93.6%)	RR 0.98 (0.92 to 1.05)	19 fewer per 1,000 (from 75 fewer to 47 more)	Moderate
Adequate color or translucency (follow-up: range 3 months to 4 years)											
11	randomized trials	not serious	not serious	not serious	not serious	none	401/408 (98.3%)	377/393 (95.9%)	RR 1.01 (0.99 to 1.02)	10 more per 1,000 (from 10 fewer to 19 more)	High
Proper surface texture or luster (follow-up: range 3 months to 10 years)											
10	randomized trials	not serious	very serious	not serious	not serious	none	310/325 (95.4%)	299/302 (99.0%)	RR 1.00 (0.96 to 1.04)	0 fewer per 1,000 (from 40 fewer to 40 more)	Low
Proper surface staining (follow-up: range 3 years to 10 years)											
4	randomized trials	not serious	not serious	not serious	not serious	none	137/138 (99.3%)	141/141 (100.0%)	RR 1.00 (0.98 to 1.03)	0 fewer per 1,000 (from 20 fewer to 30 more)	High
Retention (follow-up: range 3 months to 10 years)											
18	randomized trials	not serious	very serious	not serious	not serious	none	742/803 (92.4%)	688/786 (87.5%)	RR 1.03 (0.98 to 1.09)	26 more per 1,000 (from 18 fewer to 79 more)	Low
Absence of wear (follow-up: range 1 years to 10 years)											
6	randomized trials	not serious	not serious	not serious	not serious	none	291/295 (98.6%)	272/274 (99.3%)	RR 1.00 (0.99 to 1.01)	0 fewer per 1,000 (from 10 fewer to 10 more)	High
Proper anatomic form (follow-up: range 3 months to 10 years)											
13	randomized trials	not serious	serious	not serious	not serious	none	440/467 (94.2%)	425/435 (97.7%)	RR 0.99 (0.97 to 1.02)	10 fewer per 1,000 (from 29 fewer to 20 more)	Moderate
Absence of sensibility (follow-up: range 1 years to 6 years)											
7	randomized trials	not serious	not serious	not serious	not serious	none	372/376 (98.9%)	355/360 (98.6%)	RR 1.00 (0.99 to 1.01)	0 fewer per 1,000 (from 10 fewer to 10 more)	High
Adequate periodontal tissue (follow-up: range 3 months to 4 years)											
3	randomized trials	not serious	not serious	not serious	not serious	none	133/133 (100.0%)	136/137 (99.3%)	RR 1.00 (0.98 to 1.03)	0 fewer per 1,000 (from 20 fewer to 30 more)	High

GIC = Glass-ionomer cement; CR = Composite resin; CI = Confidence interval; RR = Risk relative

## Data Availability

The datasets used and/or analyzed during the current study are available from the corresponding author.
